# Highly efficient genome editing via 2A-coupled co-expression of two TALEN monomers

**DOI:** 10.1186/1756-0500-7-628

**Published:** 2014-09-10

**Authors:** Andrew Mariano, Li Xu, Renzhi Han

**Affiliations:** Department of Cell and Molecular Physiology, Loyola University Chicago Health Sciences Division, Maywood, IL 60153 USA; Department of Surgery, The Ohio State University Wexner Medical Center, Columbus, OH 43210 USA

**Keywords:** Gene editing, TALEN, 2A self-cleaving sequence

## Abstract

**Background:**

Transcription activator-like effector nucleases (TALENs) are a useful tool for targeted gene editing. TALEN monomers are traditionally expressed from two different plasmids. Each encodes a different TALEN arm that binds to a user-defined sequence and mediates gene editing. Expression of TALEN monomers in two separate plasmids requires co-delivery of each plasmid to the cell. Efficacy of gene editing may be increased if each monomer was transcribed from the same reading frame.

**Findings:**

We developed a TALEN scaffold which expresses both TALEN monomers from a single open reading frame in equal molar amount by linking both monomers with a 2A self-cleaving peptide sequence. This TALEN scaffold, named pTAL10, demonstrates higher levels of genome editing than co-transfected TALENs at similar levels of transfection efficiencies when analyzed for TALEN-induced small insertions and deletions.

**Conclusions:**

This protocol for gene editing using 2A-linked TALENs requires transfection of only one plasmid as compared to transfection of two separate plasmids encoding each TALEN monomers.

**Electronic supplementary material:**

The online version of this article (doi:10.1186/1756-0500-7-628) contains supplementary material, which is available to authorized users.

## Findings

### Background

Transcription activator-like effector nucleases (TALENs) are an exciting gene-editing tool that has emerged in recent years. Their DNA-binding domains are based on transcription activator-like effector (TALE) proteins from *Xanthomonas* plant pathogens [1–4]. The central repeat domain in the TALE structure mediates DNA binding with each repeat specifying one target base. The base preference of each repeat is determined by two critical, adjacent amino acids referred to as the “repeat variable di-residue” (RVD) which preferentially recognizes one of the four bases in the target site [5, 6]. This simple “two amino acids for one base” code enables rapid engineering of customized TALE repeat arrays that recognize a user-defined target sequence. Using default settings for TAL Effector Nucleotide Targeter 2.0 [7], one can identify a unique TALE-binding site for every nucleotide of target sequence—this frequency makes it a highly attractive gene editing tool. Indeed, TALEN has been used to modify genes in a number of different cell types and organisms with low off-target activity [8].

Several approaches have been taken to improve the specificity and efficiency of TALEN, including modification of FokI cleavage domain [9, 10], N-terminal and C-terminal segments flanking central repeat domains [11], and enrichment of edited cells by using fluorescence activated cell sorting (FACS) [12]. Two TALEN monomers are required to induce a double strand break in the genomic DNA of the cells, thus requiring co-delivery of both TALEN monomers into the target cells. To reduce the complication associated with co-transfection by which a portion of cells are only singly transfected, we previously [13] demonstrated that two functional TALEN monomers can be expressed from a single transcript by the use of a self-cleaving 2A peptide derived from equine rhinitis A virus (E2A; QCTNYALLKLAGDVESNPGP) or *Thosea asigna* virus (T2A; EGRGSLLTCGDVEENPGP) [14, 15]. Upon translation, the ribosome skips the synthesis of the glycyl-prolyl peptide bond between the ending glycine and proline of the 2A sequence, resulting in the release of the antecedent protein and the re-initiation of translation of the following proline amino acid [16]. This strategy ensures that each transfected cell receives both TALEN monomers at an equal molar amount, ultimately displaying higher gene editing efficiencies.

In the present study, we compared the gene editing performances of previously constructed TALENs targeting two different loci: MSTN [13] and AAVS1 locus [3]. Additionally, we provided a protocol that can help guide construction of a functional TALEN using pTAL10 scaffold.

### Results and discussion

**Figure 1:**
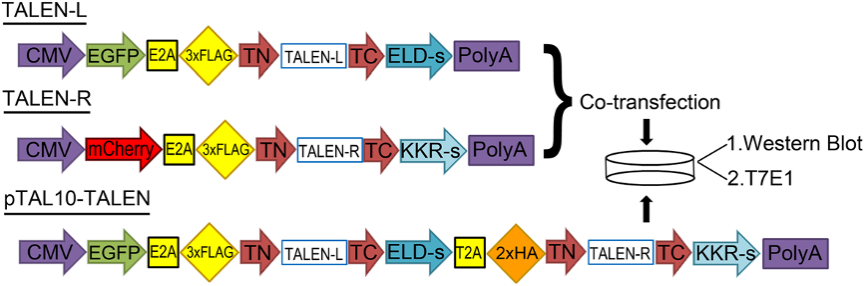
**Cartoon of relevant domains for TALEN-L (left), TALEN-R (right), and pTAL10-TALEN.** Diagram of notable regions of myostatin or AAVS1 locus targeting TALEN constructs (TALEN-L, TALEN-R, and pTAL10-TALEN) and experimental design. Monomers TALEN-L and TALEN-R contain EGFP/3xFLAG or mCherry/3xFLAG for transfection and biochemical monitoring respectively. pTAL10-TALEN contains both TALEN monomers, linked by a self-peptide cleaving sequence. Left and right monomers can be fluorescently or biochemically monitored by EGFP/3xFLAG or mCherry/2xHA tags respectively.

**Figure 2:**
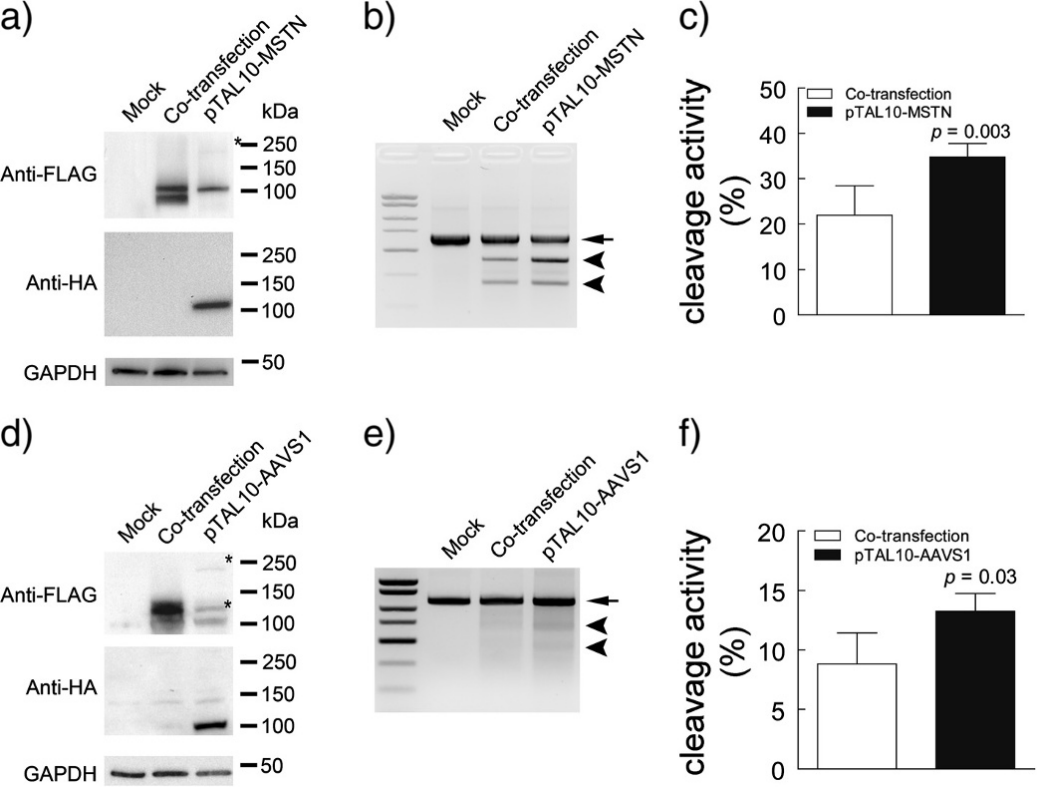
**T2A-linked TALENs demonstrate increased gene editing activity than co-transfected TALEN monomers. (a, d)** Western blotting shows proper expression of co-transfected TALENs, tagged with 3xFLAG sequence, or pTAL10-TALENs, tagged with 2xHA/3xFLAG sequences, targeting MSTN or AAVS1 respectively. Asterisk (*) depicts uncleaved TALENs arms or GFP-TALEN. GAPDH was used as a loading control. **(b, e)** Representative gel of T7E1 assay comparing gene editing activities of co-transfected TALENs and T2A-linked TALENs targeting MSTN or AAVS1 respectively. Upper band, demarcated with an arrow, represents noncleaved DNA; bottom bands, demarcated with an arrow head, represent cleaved DNA fragments. Image is representative of 4–6 biological replicates. **(c, f)** Cleavage activity of TALEN (TALEN-L/R co-transfection vs. pTAL10-TALEN) targeting MSTN or AAVS1 respectively were compared using T7E1 endonuclease assay. Results are from 4–6 biological replicates. *p* < 0.05 was considered to be significant.

We quantitatively compared the gene editing performance of our previously characterized TALENs targeting myostatin [13] expressed in one plasmid with the same TALENs expressed in two separate plasmids. HEK293 cells were transfected with either mock, co-transfection (myostatin TALEN left and right monomers in two separate plasmids), or pTAL10-MSTN (two TALEN monomers in one plasmid) (Figure 1). Western blotting showed that the TALEN monomers (~110 kDa) were faithfully expressed from either the two separate plasmids (each TALEN monomer is fused with a 3×FLAG tag) or the single plasmid linked by a T2A sequence (right TALEN monomer is fused with a 2×HA tag, whereas the left monomer is fused with a 3×FLAG tag) (Figure 2a). Consistent with previous reports that T2A mediates high self-cleavage efficiency [15], only a very small fraction of the 220 kDa full-length precursor protein was visible with a high exposure time (Figure 2a). Gene editing activity was quantified by the mismatch-sensitive T7 endonuclease I (T7E1) assay which detects the TALEN-induced insertions and deletions (indels) around the target site (Figure 2b and c). TALENs expressed from one single plasmid exhibited higher gene editing activity than co-transfection of two TALEN monomers under the same conditions (Figure 2b and c).

To test whether the improved gene editing activity is MSTN specific, we constructed another previously characterized TALENs targeting human AAVS1 locus [17]. Again, the AAVS1-TALEN monomers were faithfully expressed and properly cleaved using the pTAL10 scaffold (Figure 2d). T7E1 assay showed that AAVS1 TALENs expressed from the one-plasmid pTAL10 induced higher gene editing activity than the two separated plasmid system (Figure 2e and f).

**Figure 3:**
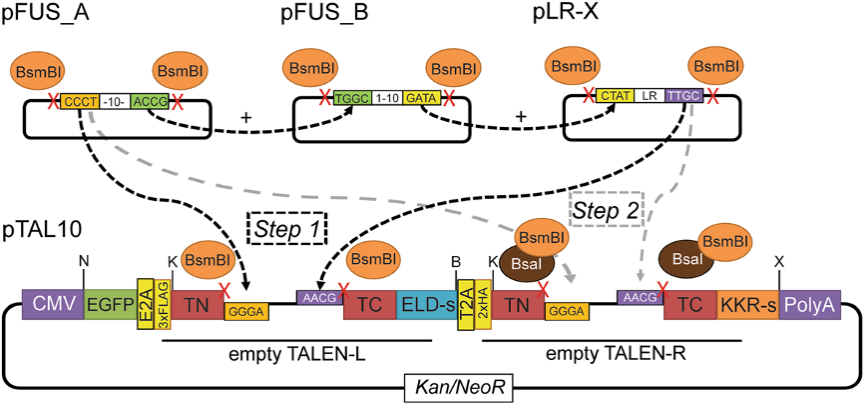
**Schematic of sequential assembly of TALE repeats into pTAL10.** pTAL10 is comprised of GFP (transfection reporter) and two TALE nuclease scaffolds denoted as “left” and “right”. TALE repeats are assembled into the left and right scaffolds sequentially by the use of BsmBI and BsaI/BsmBI restriction enzymes, respectively. The scaffold also contains kanamycin-resistant cassette for selection in bacteria and neomycin- resistant cassette for selection in mammalian cells. The listed restriction sites are: N, NheI; K, KpnI; B, BglII; X, XhoI.

To facilitate the assembly of two TALEN monomers into one plasmid, we constructed a TALEN scaffold designated as pTAL10 (Figure 3), wherein two TALEN monomers (left and right) can be sequentially assembled in a similar way as previously described [4] with some modifications. After assembly of user-defined RVD sequences in their respective pFUS backbone, these RVD sequences and last repeat sequence (pLR-X, where X represents RVD) can be ligated to the left TALEN scaffold of pTAL10 upon digestion with BsmBI (Figure 3, Step 1). To complete construction, Golden Gate assembly is repeated on the pTAL10 containing assembled left TALEN for the right monomer. Different from the previous reaction, this digestion includes both BsmBI and BsaI for unveiling appropriate sticky-ends (Figure 3, Step 2). Each TALEN monomer scaffold is based on the GoldyTALEN [18] which has increased gene editing efficiency.

In order to detect expression of each monomer uniquely by western blotting analysis, each monomer is fused with a tag at its N-terminal: 3xFLAG for left TALEN monomer, and 2xHA for the right. In addition, the TALEN monomer scaffold uses the obligate heterodimeric FokI variants (ELD-sharkey or KKR-sharkey) [13]. The two monomers and GFP (used as a reporter for transfection) are linked by the aforementioned *Thosea asigna* and equine rhinitis A virus 2A peptide sequences, respectively. We removed all BsaI and BsmBI sites outside of the TALEN scaffold in the final pTAL10 plasmid using site-directed mutagenesis so that it is fully compatible with the Golden Gate platform [4]. The map and sequence of pTAL10 as well as a detailed step-by-step protocol to assemble the TALEN monomers into this single TALEN scaffold plasmid are provided in the supplement (Additional files 1 and 2).

## Conclusion

We have generated an improved TALEN scaffold, designated as pTAL10, by utilizing a self-cleaving peptide sequence. pTAL10 demonstrates higher gene editing when compared to co-transfected TALEN-L and R due to proper co-delivery of TALE monomers. Additionally, this improved scaffold allows transfection efficiency to be fluorescently monitored, while expression of each TALEN arm to be biochemically monitored.

## Methods

### Creation of TALE nucleases

Construction of AAVS1-L, AAVS1-R, MSTN-L, and MSTN-R for co-transfection was previously described [13]. AAVS1-L/AAVS1-R and MSTN-L/MSTN-R for creation of pTAL10-AAVS1 and pTAL10-MSTN, respectively, was constructed using Golden Gate TALEN Kit 2.0 (Kit #1000000024) from Addgene. These sequences were then added to pTAL10 (Figure 2) by using BsmBI and BsmBI/BsaI digestion respectively. Further details on creation of TALE nucleases using pTAL10 scaffold is discussed below and in the supplemental protocol.

### Cell culture and transfection

HEK293 cells were cultured in DMEM supplemented with 10% FBS, 1% L-glutamine (Thermo Scientific Hyclone, Waltham, MA), and 1% Penicillin-Streptomycin (Thermo Scientific Hyclone, Waltham, MA). Transient transfection by Xfect transfection reagent (Clontech, Mountain View, CA) was done according to manufacturer’s recommendation. In short, HEK293 cells for each downstream application (genomic DNA or protein extraction) were seeded in a 6-well plate until 60-70% confluency. Thereafter, 5 μg of total DNA (pTAL10-TALEN single plasmid transfection, or TALEN-L/TALEN-R co-transfection) in nanoparticle complex solution was added to the culture medium dropwise, followed by replacement of medium 16–20 hours post transfection. After 72 hours, either genomic DNA or protein was extracted from these cell lines. Transfection efficiency was determined by fluorescence microscopy analysis: 1) the average percentage of mCherry- or GFP-positive cells for co-transfection or 2) the percentage of GFP-positive cells for pTAL10-TALENs.

### Western blot

HEK293 transfected with single pTAL10-MSTN or co-transfection with MSTN-L and MSTN-R were lysed 48–72 hours post transfection with cold RIPA buffer supplemented with protease inhibitors (1 mM PMSF and 1 mM Benzamidine). Protein concentration was determined by using Bradford Protein Assay Kit (IBI Scientific, Peosta, IA). 20 μg of protein were resolved on a 4-20% Mini-PROTEAN TGX Precast Gel (Bio-Rad Laboratories, Hercules, CA) and transferred onto a PVDF membrane (Millipore, Billerica, MA). Immunoblotting was done with the following antibodies: mouse monoclonal anti-FLAG (F3165; Sigma-Aldrich, Saint Louis, MO), polyclonal anti-HA-Tag antibody (Clontech, Mountain View, CA), mouse monoclonal anti-GAPDH (Millipore, Billerica, MA), and HRP conjugated goat anti-mouse IgG (Millipore, Billerica, MA). Membranes were developed using ECL 2 Western Blotting Substrate (Pierce Biotechnology, Rockford, IL) and images were captured using ChemiDoc XRS + system (Bio-Rad Laboratories, Hercules, CA).

### T7E1 mismatch-detection assay

Cleavage activity of AAVS1 and MSTN TALENs, as measured by small deletion/insertion mutations (indels), were quantified by mismatch-recognizing T7E1 as described previously [13]. Briefly, cells transfected with respective TALENs had genomic DNA extracted 48–72 hours post-transfection. Amplification was achieved by using AccuPrime Pfx (Invitrogen, Carlsbad, CA). PCR primers were designed to amplify site surrounding human AAVS1 or human MSTN locus. Specifically, the primer pair for human MSTN binds to Intron 1–2 (hGDF8-F1, 5′-TGGAGGGGTTTTGTTAATGG-3′) and Intron 2–3 (hGDF8-F2, 5′-TATTGGGTACAGGGCTACCG-3′). The primer pair used for AAVS1 targets the following sites: AS1L-833 F (5′- TTCAGGTTCCGTCTTCCTCCACTC – 3′) and AS1l-1998R (5′- AACTGACGCACGGAGGAACAAT-3′). The DNA fragment was purified by using Wizard SV Gel and PCR Clean-Up (Promega, Madison, WI), followed by measurement of DNA concentration by using NanoDrop 2000c spectrophotometer (Thermo Fisher Scientific, Wilmingdon, DE). 600 ng of DNA for MSTN targeting TALEN or 200 ng of DNA for AAVS1 in NEBuffer 2 (New England BioLabs, Ipswich, MA) was then denatured at 95°C for 10 minutes, and reannealed slowly using the following temperature program to form DNA heteroduplex if NHEJ occurred: 90 cycles of 95-59°C with a 0.4°C decrease per cycle for 20 seconds, 90 cycles of 59 to 32°C with a 0.3°C decrease per cycle for 20 seconds, and 20 cycles of 32 to 26°C with a 0.3°C decrease per cycle for 20 seconds. 0.5 uL T7 Endonuclease I (T7E1; New England BioLabs, Ipswich, MA) was added to the reannealed DNA samples, followed by incubation for 30 minutes. DNA samples were then subjected to electrophoresis on a 2% TAE agarose gel containing Gel-Red (Biotium, Mayward, CA). The gels were imaged using ChemiDoc XRS + system (Bio-Rad Laboratories, Hercules, CA), and densiometry of DNA bands were quantified using ImageJ software (NIH, Frederick, MD). Mutation frequencies were calculated using the formula: fractional modification = 1-(1-(total densiometry of fraction cleaved/total densiometry))^0.5^ as described [18].

### Fluorescence microscopy

Fluorescence and bright-field images were taken as previously mentioned [13]. In short, images were taken using an inverted Nikon Ti-E microscope equipped with a Xenon lamp (Hamamatsu Photonics Systems, Bridgewater, NJ), a 40 × 1.30 NA objective (Nikon, Tokyo, Japan), and an Evolve 512 EMCCD camera (Photometrics, Pleasanton, CA). The EMCCD camera was cooled to -80°C during imaging. Images were documented and analyzed using NIS-Elements Advanced Research software package (Nikon, Tokyo, Japan).

### Statistical analysis

All data were expressed as mean ± SEM. Statistical differences were determined by unpaired Student’s t-test for two groups using GraphPad Prism 5.0. Statistical significance was defined to be *p* < 0.05.

## Electronic supplementary material

Additional file 1: **The sequence of pTAL10.** (DOCX 95 KB)

Additional file 2: **An improved self-cleaving peptide-linked TALEN scaffold for efficient genome editing.** (DOC 68 KB)

## Abbreviations

PCR: Polymerase chain reaction
TALEN: Transcription activator-like effector nuclease
GFP: Green fluorescent protein
RVD: Repeat-variable di-residue
MSTN: Myostatin
HA: Hemagglutinin
T7E1: T7 endonuclease I.
