# Supplementary material for: Highly efficient genome editing via 2A-coupled co-expression of two TALEN monomers
Source: BMC Res Notes. 2014 Sep 10;7:628. doi: 10.1186/1756-0500-7-628 (PMC4167141; doi:10.1186/1756-0500-7-628)

**Supplemental Information:**

1. The sequence of pTAL10:

TAGTTATTAATAGTAATCAATTACGGGGTCATTAGTTCATAGCCCATATATGGAGTTCCGCGTTACATAACTTACGGTAAATGGCCCGCCTGGCTGACCGCCCAACGACCCCCGCCCATTGACGTCAATAATGACGTATGTTCCCATAGTAACGCCAATAGGGACTTTCCATTGACGTCAATGGGTGGAGTATTTACGGTAAACTGCCCACTTGGCAGTACATCAAGTGTATCATATGCCAAGTACGCCCCCTATTGACGTCAATGACGGTAAATGGCCCGCCTGGCATTATGCCCAGTACATGACCTTATGGGACTTTCCTACTTGGCAGTACATCTACGTATTAGTCATCGCTATTACCATGGTGATGCGGTTTTGGCAGTACATCAATGGGCGTGGATAGCGGTTTGACTCACGGGGATTTCCAAGTCTCCACCCCATTGACGTCAATGGGAGTTTGTTTTGGCACCAAAATCAACGGGACTTTCCAAAATGTCGTAACAACTCCGCCCCATTGACGCAAATGGGCGGTAGGCGTGTACGGTGGGAGGTCTATATAAGCAGAGCTGGTTTAGTGAACCGTCAGATCCGCTAGCGCTACCGGTCGCCACCATGGTGAGCAAGGGCGAGGAGCTGTTCACCGGGGTGGTGCCCATCCTGGTCGAGCTGGACGGCGACGTAAACGGCCACAAGTTCAGCGTGTCCGGCGAGGGCGAGGGCGATGCCACCTACGGCAAGCTGACCCTGAAGTTCATCTGCACCACCGGCAAGCTGCCCGTGCCCTGGCCCACCCTCGTGACCACCCTGACCTACGGCGTGCAGTGCTTCAGCCGCTACCCCGACCACATGAAGCAGCACGACTTCTTCAAGTCCGCCATGCCCGAAGGCTACGTCCAGGAGCGCACCATCTTCTTCAAGGACGACGGCAACTACAAGACCCGCGCCGAGGTGAAGTTCGAGGGCGACACCCTGGTGAACCGCATCGAGCTGAAGGGCATCGACTTCAAGGAGGACGGCAACATCCTGGGGCACAAGCTGGAGTACAACTACAACAGCCACAACGTCTATATCATGGCCGACAAGCAGAAGAACGGCATCAAGGTGAACTTCAAGATCCGCCACAACATCGAGGACGGCAGCGTGCAGCTCGCCGACCACTACCAGCAGAACACCCCCATCGGCGACGGCCCCGTGCTGCTGCCCGACAACCACTACCTGAGCACCCAGTCCGCCCTGAGCAAAGACCCCAACGAGAAGCGCGATCACATGGTCCTGCTGGAGTTCGTGACCGCCGCCGGGATCACTCTCGGCATGGACGAGCTGTACAAGCAATGTACTAACTACGCTTTGTTGAAACTCGCTGGCGATGTTGAAAGTAACCCCGGTCCTGAATTCATGGACTACAAAGACCATGACGGTGATTATAAAGATCATGACATCGATTACAAGGATGACGATGACAAGATGGCCCCCAAGAAGAAGAGGAAGGTGGGCATTCACGGGGTACCtATGGTGGATCTACGCACGCTCGGCTACAGTCAGCAGCAGCAAGAGAAGATCAAACCGAAGGTGCGTTCGACAGTGGCGCAGCACCACGAGGCACTGGTGGGCCATGGGTTTACACACGCGCACATCGTTGCGCTCAGCCAACACCCGGCAGCGTTAGGGACCGTCGCTGTCACGTATCAGCACATAATCACGGCGTTGCCAGAGGCGACACACGAAGACATCGTTGGCGTCGGCAAACAGTGGTCCGGCGCACGCGCCCTGGAGGCCTTGCTCACGGATGCGGGGGAGTTGAGAGGTCCGCCGTTACAGTTGGACACAGGCCAACTTGTGAAGATTGCAAAACGTGGCGGCGTGACCGCAATGGAGGCAGTGCATGCATCGCGCAATGCACTGACGGGTGCTC**ccctggagacgTCGACcgtctccaacg**acCACCTCGTCGCCTTGGCCTGCCTCGGCGGACGTCCTGCCATGGATGCAGTGAAAAAGGGATTGCCGCACGCGCCGGAATTGATCAGAAGAGTCAATCGCCGTATTGGCGAACGCACGTCCCATCGCGTTGCCGGATCCCAGCTGGTCAAAAGTGAACTGGAGGAGAAGAAATCTGAACTTCGTCATAAATTGAAATATGTGCCTCATGAATATATTGAATTAATTGAAATTGCCAGAAATcCCACTCAGGATAGgATTCTTGAAATGAAGGTAATGGAATTTTTTATGAAAGTTTATGGATATAGAGGTgAACATTTGGGTGGATCAAGGAAACCGGACGGAGCAATTTATACTGTCGGATCTCCTATTGATTACGGTGTGATCGTGGATACTAAAGCTTATAGCGGAGGTTATAATCTGCCAATTGGCCAAGCAGATGAAATGgAACGATATGTCGAAGAAAATCAAACACGAgACAAACATcTCAACCCTAATGAATGGTGGAAAGTCTATCCATCTTCTGTAACGGAATTTAAGTTTTTATTTGTGAGTGGTCACTTTAAAGGAAACTACAAAGCTCAGCTTACACGATTAAATCATATCACTAATTGTAATGGAGCTGTTCTTAGTGTAGAAGAGCTTTTAATTGGTGGAGAAATGATTAAAGCCGGCACATTAACCTTAGAGGAAGTGAGAaGGAAATTTAATAACGGCGAGATAAACTTTagatctGGCGGCGGAGAGGGCAGAGGAAGTCTTCTAACATGCGGTGACGTGGAGGAGAATCCCGGCCCTAGGATGtacccatacgatgttccagattacgctATGtacccatacgatgttccagattacgctATGGCCCCCAAGAAGAAGAGGAAGGTGGGCATTCACGGGGTACCtATGGTGGATCTACGCACGCTCGGCTACAGTCAGCAGCAGCAAGAGAAGATCAAACCGAAGGTGCGTTCGACAGTGGCGCAGCACCACGAGGCACTGGTGGGCCATGGGTTTACACACGCGCACATCGTTGCGCTCAGCCAACACCCGGCAGCGTTAGGGACCGTCGCTGTCACGTATCAGCACATAATCACGGCGTTGCCAGAGGCGACACACGAAGACATCGTTGGCGTCGGCAAACAGTGGTCCGGCGCACGCGCCCTGGAGGCCTTGCTCACGGATGCGGGGGAGTTGAGAGGTCCGCCGTTACAGTTGGACACAGGCCAACTTGTGAAGATTGCAAAACGTGGCGGCGTGACCGCAATGGAGGCAGTGCATGCATCGCGCAATGCACTGACGGGTGCTC**ccctggagaccGTCGACggtctccaacg**acCACCTCGTCGCCTTGGCCTGCCTCGGCGGACGTCCTGCCATGGATGCAGTGAAAAAGGGATTGCCGCACGCGCCGGAATTGATCAGAAGAGTCAATCGCCGTATTGGCGAACGCACGTCCCATCGCGTTGCCGGATCCCAGCTGGTCAAAAGTGAACTGGAGGAGAAGAAATCTGAACTTCGTCATAAATTGAAATATGTGCCTCATGAATATATTGAATTAATTGAAATTGCCAGAAATcCCACTCAGGATAGgATTCTTGAAATGAAGGTAATGGAATTTTTTATGAAAGTTTATGGATATAGAGGTgAACATTTGGGTGGATCAAGGAAACCGGACGGAGCAATTTATACTGTCGGATCTCCTATTGATTACGGTGTGATCGTGGATACTAAAGCTTATAGCGGAGGTTATAATCTGCCAATTGGCCAAGCAGATGAAATGCAACGATATGTCaAgGAAAATCAAACACGAAACAAACATATCAACCCTAATGAATGGTGGAAAGTCTATCCATCTTCTGTAACGGAATTTAAGTTTTTATTTGTGAGTGGTCACTTTAAAGGAAACTACAAAGCTCAGCTTACACGATTAAATCgTAagACTAATTGTAATGGAGCTGTTCTTAGTGTAGAAGAGCTTTTAATTGGTGGAGAAATGATTAAAGCCGGCACATTAACCTTAGAGGAAGTGAGAaGGAAATTTAATAACGGCGAGATAAACTTTTAACTCGAGTGATCCACCGGATCTAGATAACTGATCATAATCAGCCATACCACATTTGTAGAGGTTTTACTTGCTTTAAAAAACCTCCCACACCTCCCCCTGAACCTGAAACATAAAATGAATGCAATTGTTGTTGTTAACTTGTTTATTGCAGCTTATAATGGTTACAAATAAAGCAATAGCATCACAAATTTCACAAATAAAGCATTTTTTTCACTGCATTCTAGTTGTGGTTTGTCCAAACTCATCAATGTATCTTAACGCGTAAATTGTAAGCGTTAATATTTTGTTAAAATTCGCGTTAAATTTTTGTTAAATCAGCTCATTTTTTAACCAATAGGCCGAAATCGGCAAAATCCCTTATAAATCAAAAGAATAGACCGAGATAGGGTTGAGTGTTGTTCCAGTTTGGAACAAGAGTCCACTATTAAAGAACGTGGACTCCAACGTCAAAGGGCGAAAAACCGTCTATCAGGGCGATGGCCCACTACGTGAACCATCACCCTAATCAAGTTTTTTGGGGTCGAGGTGCCGTAAAGCACTAAATCGGAACCCTAAAGGGAGCCCCCGATTTAGAGCTTGACGGGGAAAGCCGGCGAACGTGGCGAGAAAGGAAGGGAAGAAAGCGAAAGGAGCGGGCGCTAGGGCGCTGGCAAGTGTAGCGGTCACGCTGCGCGTAACCACCACACCCGCCGCGCTTAATGCGCCGCTACAGGGCGCGTCAGGTGGCACTTTTCGGGGAAATGTGCGCGGAACCCCTATTTGTTTATTTTTCTAAATACATTCAAATATGTATCCGCTCATGAGACAATAACCCTGATAAATGCTTCAATAATATTGAAAAAGGAAGAGTCCTGAGGCGGAAAGAACCAGCTGTGGAATGTGTGTCAGTTAGGGTGTGGAAAGTCCCCAGGCTCCCCAGCAGGCAGAAGTATGCAAAGCATGCATCTCAATTAGTCAGCAACCAGGTGTGGAAAGTCCCCAGGCTCCCCAGCAGGCAGAAGTATGCAAAGCATGCATCTCAATTAGTCAGCAACCATAGTCCCGCCCCTAACTCCGCCCATCCCGCCCCTAACTCCGCCCAGTTCCGCCCATTCTCCGCCCCATGGCTGACTAATTTTTTTTATTTATGCAGAGGCCGAGGCCGCCTCGGCCTCTGAGCTATTCCAGAAGTAGTGAGGAGGCTTTTTTGGAGGCCTAGGCTTTTGCAAAGATCGATCAAGAGACAGGATGAGGATCGTTTCGCATGATTGAACAAGATGGATTGCACGCAGGTTCTCCGGCCGCTTGGGTGGAGAGGCTATTCGGCTATGACTGGGCACAACAGACAATCGGCTGCTCTGATGCCGCCGTGTTCCGGCTGTCAGCGCAGGGGCGCCCGGTTCTTTTTGTCAAGACCGACCTGTCCGGTGCCCTGAATGAACTGCAAGACGAGGCAGCGCGGCTATCGTGGCTGGCCACGACGGGCGTTCCTTGCGCAGCTGTGCTCGACGTTGTCACTGAAGCGGGAAGGGACTGGCTGCTATTGGGCGAAGTGCCGGGGCAGGATCTCCTGTCATCTCACCTTGCTCCTGCCGAGAAAGTATCCATCATGGCTGATGCAATGCGGCGGCTGCATACGCTTGATCCGGCTACCTGCCCATTCGACCACCAAGCGAAACATCGCATCGAGCGAGCACGTACTCGGATGGAAGCCGGTCTTGTCGATCAGGATGATCTGGACGAAGAGCATCAGGGGCTCGCGCCAGCCGAACTGTTCGCCAGGCTCAAGGCGAGCATGCCCGACGGCGAGGATCTCGTCGTGACCCATGGCGATGCCTGCTTGCCGAATATCATGGTGGAAAATGGCCGCTTTTCTGGATTCATCGACTGTGGCCGGCTGGGTGTGGCGGACCGCTATCAGGACATAGCGTTGGCTACCCGTGATATTGCTGAAGAGCTTGGCGGCGAATGGGCTGACCGCTTCCTCGTGCTTTACGGTATCGCCGCTCCCGATTCGCAGCGCATCGCCTTCTATCGCCTTCTTGACGAGTTCTTCTGAGCGGGACTCTGGGGTTCGAAATGACCGACCAAGCGACGCCCAACCTGCCATCACGAGATTTCGATTCCACCGCCGCCTTCTATGAAAGGTTGGGCTTCGGAATCGTTTTCCGGGACGCCGGCTGGATGATCCTCCAGCGCGGGGATCTCATGCTGGAGTTCTTCGCCCACCCTAGGGGGAGGCTAACTGAAACACGGAAGGAGACAATACCGGAAGGAACCCGCGCTATGACGGCAATAAAAAGACAGAATAAAACGCACGGTGTTGGGTCGTTTGTTCATAAACGCGGGGTTCGGTCCCAGGGCTGGCACTCTGTCGATACCCCACCGAGACaCCATTGGGGCCAATACGCCCGCGTTTCTTCCTTTTCCCCACCCCACCCCCCAAGTTCGGGTGAAGGCCCAGGGCTCGCAGCCAACGTCGGGGCGGCAGGCCCTGCCATAGCCTCAGGTTACTCATATATACTTTAGATTGATTTAAAACTTCATTTTTAATTTAAAAGGATCTAGGTGAAGATCCTTTTTGATAATCTCATGACCAAAATCCCTTAACGTGAGTTTTCGTTCCACTGAGCGTCAGACCCCGTAGAAAAGATCAAAGGATCTTCTTGAGATCCTTTTTTTCTGCGCGTAATCTGCTGCTTGCAAACAAAAAAACCACCGCTACCAGCGGTGGTTTGTTTGCCGGATCAAGAGCTACCAACTCTTTTTCCGAAGGTAACTGGCTTCAGCAGAGCGCAGATACCAAATACTGTCCTTCTAGTGTAGCCGTAGTTAGGCCACCACTTCAAGAACTCTGTAGCACCGCCTACATACCTCGCTCTGCTAATCCTGTTACCAGTGGCTGCTGCCAGTGGCGATAAGTCGTGTCTTACCGGGTTGGACTCAAGACGATAGTTACCGGATAAGGCGCAGCGGTCGGGCTGAACGGGGGGTTCGTGCACACAGCCCAGCTTGGAGCGAACGACCTACACCGAACTGAGATACCTACAGCGTGAGCTATGAGAAAGCGCCACGCTTCCCGAAGGGAGAAAGGCGGACAGGTATCCGGTAAGCGGCAGGGTCGGAACAGGAGAGCGCACGAGGGAGCTTCCAGGGGGAAACGCCTGGTATCTTTATAGTCCTGTCGGGTTTCGCCACCTCTGACTTGAGCGTCGATTTTTGTGATGCTCGTCAGGGGGGCGGAGCCTATGGAAAAACGCCAGCAACGCGGCCTTTTTACGGTTCCTGGCCTTTTGCTGGCCTTTTGCTCACATGTTCTTTCCTGCGTTATCCCCTGATTCTGTGGATAACCGTATTACCGCCATGCAT

ELD-s

Left scaffold

3xFLAG/NLS

E2A

EGFP

T2A

2xHA/NLS

Right scaffold

KKR-s

2. The map of pTAL10:


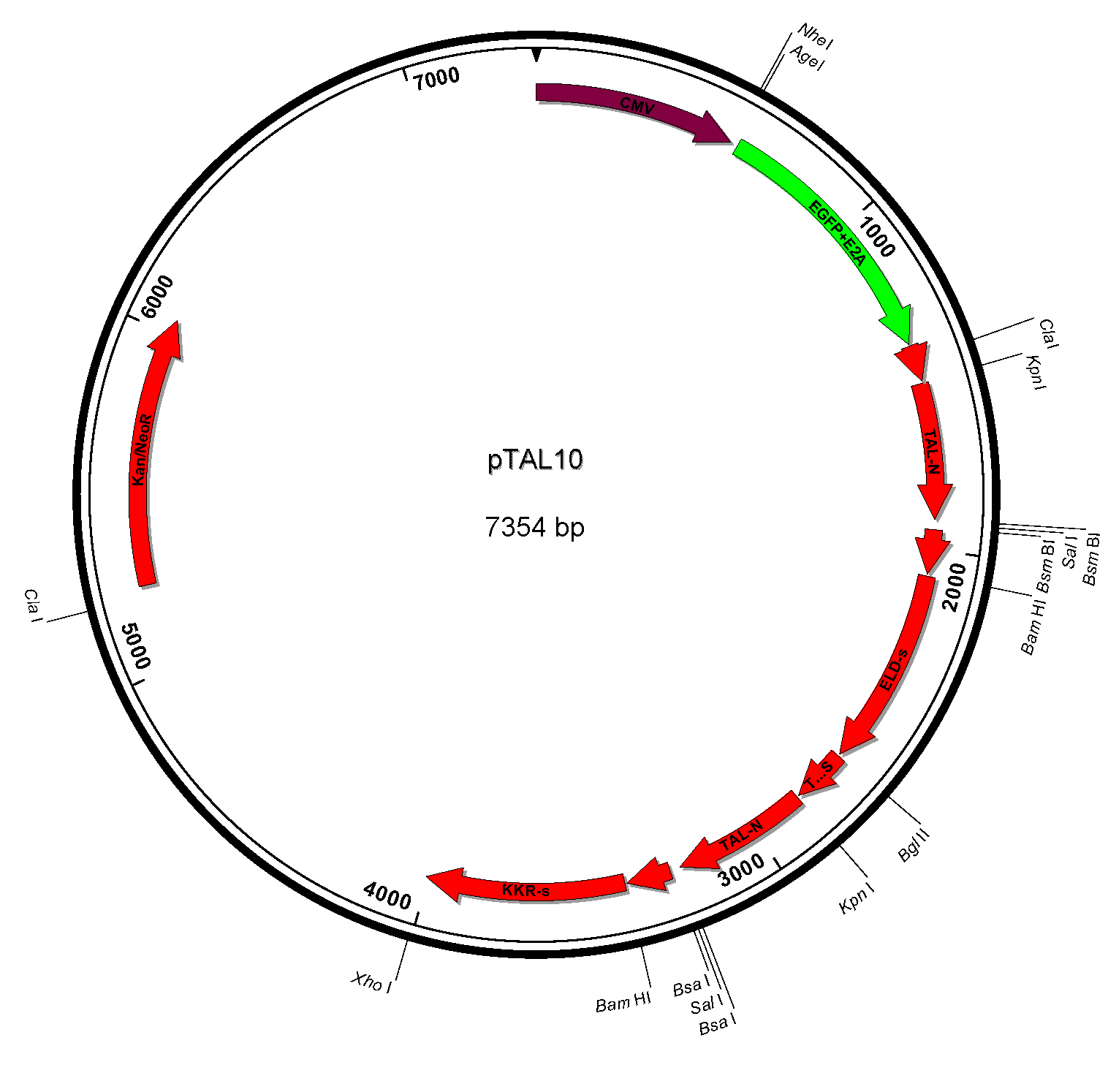

Supplement: Supplementary file 1 — Additional file 1: The sequence of pTAL10. (DOCX 95 KB) [file 13104_2013_3154_MOESM1_ESM.docx]
