# Supplementary material for: Highly efficient genome editing via 2A-coupled co-expression of two TALEN monomers
Source: BMC Res Notes. 2014 Sep 10;7:628. doi: 10.1186/1756-0500-7-628 (PMC4167141; doi:10.1186/1756-0500-7-628)
Supplement: Supplementary file 2 — Additional file 2: An improved self-cleaving peptide-linked TALEN scaffold for efficient genome editing. (DOC 68 KB) [file 13104_2013_3154_MOESM2_ESM.doc]

**2A-linked TALEN assembly protocol**

**PROTOCOL FOR:**

**An improved self-cleaving peptide-linked TALEN scaffold for efficient genome editing**

Andrew Mariano, Li Xu and Renzhi Han

Department of Cell and Molecular Physiology, Loyola University Chicago Health Science Division, Maywood, IL 60153, United States

**LEGEND**

***ATTENTION***

*** *HINT***

**REAGENTS**

Golden Gate TALEN 2.0 (TALEN Kit #1000000024, Addgene)

AccuPrime *Pfx* (Cat#: 12344-024; Invitrogen, Carlsbad, CA)

10X AccuPrime *Pfx* Reaction Mix (Cat#: 12344-024; Invitrogen, Carlsbad, CA)

T4 DNA Ligase (Cat#: M0202; New England BioLabs, Ipswich, MA)

T4 DNA Ligase Reaction Buffer (Cat#: M0202; New England BioLabs, Ipswich, MA)

BSA, Molecular Biology Grade (Cat#: B9000; New England BioLabs, Ipswich, MA)

BsmBI (Cat#: R0580; New England BioLabs, Ipswich, MA)

BsaI (Cat#: R0535; New England BioLabs, Ipswich, MA)

NheI (Cat#: R0131; New England BioLabs, Ipswich, MA)

BglII (Cat#: R0144; New England BioLabs, Ipswich, MA)

XhoI (Cat#: R0146; New England BioLabs, Ipswich, MA)

T7 Endonuclease I (T7E1; Cat#: M0302; New England BioLabs, Ipswich, MA)

**PRIMERS**

**Name**

| **Name** | **Primer Sequence (5’🡪3’)** | **Additional Notes** |
| --- | --- | --- |
| pCR8_F1 | ttgatgcctggcagttccct | Used for confirming correct pFUS vectors |
| pCR8_R1 | cgaaccgaacaggcttatgt | Used for confirming correct pFUS vectors |

**PROCEDURE**

**(I) Assemble TALENs into pTAL10**

1. Follow the Golden Gate TALEN assembly protocol (<http://www.addgene.org/static/cms/files/Golden_Gate_TALEN_assembly_v6.pdf>) for **Day 1** and **Day 2** to assemble, screen and miniprep.

| **If TALEN length is between 12-21…** | **If TALEN length is between 22-31…** |
| --- | --- |
| pFUS_A with first 10 repeats cloned (A)  pFUS_B with 11-(N-1) repeats cloned (B) | pFUS_A30A with first 10 repeats cloned (A1)  pFUS_A30B with second 10 repeats cloned (A2)  pFUS_B with 21-(N-1) repeats cloned (B) |

2. *Optional: restriction digestion and/or sequencing:*

Use enzymes AflII and XbaI (same for all destination vectors) to cut out the array of fused repeats: 1048bp for pFUS_A vectors, different sizes depending on number of repeats cloned for pFUS_B vectors; and/or sequence with primers pCR8_F1, pCR8_R1

3. Mix golden gate reaction #2 to assemble the first (“Left”) TALEN monomer on pTAL10

| Reagent | Amount |
| --- | --- |
| pTAL10 | 75ng |
| Plasmids containing left TALE array | 150ng each |
| Plasmid containing respective pLR vector | 150ng |
| BsmBI (10U/µl) | 1µl |
| BSA (10 mg/mL) | 2µl |
| T4 DNA Ligase Buffer | 2µl |
| T4 DNA Ligase (2000U/µl) | 1µl |
| Nuclease-free water | Fill to 20µl |

4. Subject the above reaction mixture to the following protocol within a thermocycler:

| Temperature | Time | Cycles |
| --- | --- | --- |
| 37°C | 5” |  |
| 16°C | 10” | x10 |
| 50°C | 20” |  |
| 80°C | 5” |  |

5. Transform your competent cells (use 5µl of the reaction)

Note: Plasmid-Safe nuclease treatment is not necessary in this case, because the final destination vector has no homology with the inserted repeats.

6. Plate on **Kanamycin plates** (*note: this is different from the Voytas lab protocol*).

7. Pick 1-4 colonies, grow up an over-night culture and miniprep the pTAL10 vectors containing full-length first TALEN monomer

8. Screen the minipreps by restriction digestion with NheI and BglII. Correct clones should produce a band over 3kb depending on the number of repeats you have assembled.

9. Mix golden gate reaction #3 (to assemble the second TALEN monomer on pTAL10 with 1st TALEN monomer)

| Reagent | Amount |
| --- | --- |
| Each pFUS vector | 150ng |
| pTAL10 plasmid containing left TALE array | 75ng |
| Plasmid containing respective pLR vector | 150ng |
| BsmBI (10U/µl) | 1µl |
| **BsaI (10U/µl)** | **1µl** |
| BSA (10 mg/mL) | 2µl |
| T4 DNA Ligase Buffer | 2µl |
| T4 DNA Ligase (2000U/µl) | 1µl |
| Nuclease-free water | Fill to 20µl |

10. Repeat steps 4-7.

11. Screen the minipreps by restriction digestion with BglII and XhoI. Correct clones should produce a band over 3kb depending on the number of repeats you have assembled. Your pair of TALENs in a single plasmid are ready to use in a mammalian cell line.

**(II) Assay for gene editing activity**

12. Transfect or electroporate the TALEN plasmid and a control plasmid (i.e. the empty pTAL10) into a suitable cell line (i.e. HEK293 cells)

13. 48-72 hours post transfection, harvest the cells and divide into two sets. One set is used for genomic DNA extraction and the other for protein extraction.

14. Design a pair of PCR primers to amplify the genomic DNA containing the TALEN target site and run a 50 µl PCR reaction using a high fidelity PCR kit with the genomic DNA extracted from the TALEN-transfected cells and control cells:

| Reagent | Amount |
| --- | --- |
| Genomic DNA | 200ng |
| Accuprime *Pfx* (2.5U/µl) | 1µl |
| 10X Accuprime *Pfx* Reaction Mix | 5µl |
| Forward Primer (10µM) | 1.5µl |
| Reverse Primer (10µM) | 1.5µl |
| dNTP (2.5mM) | 2.5µl |
| Nuclease-free water | Fill to 50µl |

If possible, design the PCR primers so that 1) the amplicon is about 600 to 1000 bp, and 2) two cleaved bands of distinctly different sizes can be observed.

Because the downstream T7E1 assay is highly sensitive to mismatched DNA, it is imperative that high fidelity PCR polymerase is used. Improper polymerase can result in T7E1 cleavage of PCR product amplified from mock-transfected/electroporated cells.

15. Analyze the PCR products on 1% TAE agarose gel

16. Gel extract and purify the correct PCR product, resuspend the DNA in 30 µl 1x NEB Buffer 2 or 2.1.

17. Treat the purified PCR product using the following program in a PCR cycler to facilitate the formation of heteroduplex:

| Temperature | Time | Cycles |
| --- | --- | --- |
| 95°C | 10” |  |
| 95-59°C | 20s | x90, 0.4°C decrease per cycle |
| 59-32°C | 20s | x90, 0.3°C decrease per cycle |
| 32-26°C | 20s | x20, 0.3°C decrease per cycle |

18. Treat each DNA sample with 0.5µl T7 endonuclease I (10U/µl, NEB) for 45 minutes at 37°C. * Amount of DNA can vary between 300ng-1000ng. Lower amounts of DNA require smaller incubation time to prevent non-specific cleaving. For 500ng, 45 minutes is sufficient.

19. Analyze the DNA samples on a 2% TAE agarose gel.

* You can also use high resolution polyacrylamide gel electrophoresis to analyze the DNA samples. If you are also use TAE agarose gel, it is better to use higher percentage (i.e. 2%) so that the cleaved bands can be well discerned.

20. Image the gel using ChemiDoc XRS+ system (Bio-Rad Laboratories, Hercules, CA), and quantify the gel bands using ImageJ software (NIH, Frederick, MD). Mutation frequencies can be calculated using the formula: fractional modification = 1-(1-(total densiometry of fraction cleaved/total densiometry))^0.5^ as described (1).

1.**Miller, J.C., M.C. Holmes, J. Wang, D.Y. Guschin, Y.L. Lee, I. Rupniewski, C.M. Beausejour, A.J. Waite, et al.** 2007. An improved zinc-finger nuclease architecture for highly specific genome editing. Nat Biotechnol *25*:778-785.
